# Measurement strategy and statistical power in studies assessing gait stability and variability in older adults

**DOI:** 10.1007/s40520-015-0390-8

**Published:** 2015-06-07

**Authors:** Marcel J. P. Toebes, Marco J. M. Hoozemans, Svend Erik Mathiassen, Joost Dekker, Jaap H. van Dieën

**Affiliations:** MOVE Research Institute Amsterdam, Faculty of Human Movement Sciences, VU University Amsterdam, Van der Boechorststraat 7-9, 1081BT Amsterdam, The Netherlands; Centre for Musculoskeletal Research, Department of Occupational and Public Health Sciences, University of Gävle, Gävle, Sweden; Department of Rehabilitation Medicine, VU University Medical Center, EMGO Institute for Health and Care Research, Amsterdam, The Netherlands

**Keywords:** Measurement design, Gait variability, Local dynamic stability, Walking, Between-day variance, Within-day variance

## Abstract

**Background:**

Gait variability and stability measures might be useful to assess gait quality changes after fall prevention programs. However, reliability of these measures appears limited.

**Aims:**

The objective of the present study was to assess the effects of measurement strategy in terms of numbers of subjects, measurement days and measurements per day on the power to detect relevant changes in gait variability and stability between conditions among healthy elderly.

**Methods:**

Sixteen healthy older participants [65.6 (SD 5.9) years], performed two walking trials on each of 2 days. Required numbers of subjects to obtain sufficient statistical power for comparisons between conditions within subjects (paired, repeated-measures designs) were calculated (with confidence intervals) for several gait measures and for different numbers of trials per day and for different numbers of measurement days.

**Results:**

The numbers of subjects required to obtain sufficient statistical power in studies collecting data from one trial on 1 day in each of the two compared conditions ranged from 7 to 13 for large differences but highly correlated data between conditions, up to 78–192 for data with a small effect and low correlation.

**Discussion:**

Low correlations between gait parameters in different conditions can be assumed and relatively small effects appear clinically meaningful. This implies that large numbers of subjects are generally needed.

**Conclusion:**

This study provides the analysis tools and underlying data for power analyses in studies using gait parameters as an outcome of interventions aiming to reduce fall risk.

## Introduction

A large proportion of falls in older adults occurs during locomotion [1–3]. These falls are often attributed to a decreased quality of gait, due to age-related, peripheral [4] and central [5] impairments. Gait variability and local dynamic stability have received much attention as indicators of fall-related measures of gait quality [6, 7] and several studies have confirmed that these parameters are, indeed, related to fall risk [8–13]. Although ultimately the ability to predict actual fall risk would remain to be shown, the use of gait quality measures as outcome variables in intervention studies might allow faster iterative development of fall prevention programs, as actual fall risk by gathering fall incidence data requires a long follow-up period. While reliability of gait variability and stability estimates can to some extent be improved by treadmill walking to collect data from a large number of strides [14–17], a recent study indicated that reliability between sessions is still only moderate [18]. The statistical consequences of limited test–retest reliability can be overcome by adjusting the measurement strategy, but previous reports do not allow inferences on optimal measurement strategies. In studies investigating differences in gait quality between conditions in a population, the optimal measurement strategy, in terms of the number of subjects and the number of measurements per subject, depends on the variance of the gait parameters between and within subjects.

The first and main aim of this study was to estimate between- and within-subject variance components of gait variability and stability measures in treadmill walking, to allow estimation of the number of subjects necessary to obtain sufficient statistical power in studies that are aimed at detecting relevant differences between conditions in a repeated-measures design using subjects as their own controls. The second aim was to determine how the number of measurement days or measurements per day (i.e., the within-subject data collection strategy) influences the required numbers of subjects to detect differences between conditions with sufficient statistical power.

## Materials and methods

### Subjects

Sixteen older subjects [*n*_female_ = 9, *n*_male_ = 7, mean age 65.6 (SD 5.9) years, mean weight 77.5 (SD 15.3) kg, mean height 1.74 (SD 0.09) m], without physical impairments interfering with their walking ability, participated in this study. All subjects gave informed written consent. The ethics committee of the Faculty of Human Movement Sciences, VU University Amsterdam approved the experimental protocol in accordance with the Declaration of Helsinki.

### Study design

Time series of 5 min of treadmill walking at 3.0 km h^−1^ were collected during four trials (two trials on each of 2 days). In between the walking trials, subjects performed a 15-min trial of perturbed walking at 3.0 km h^−1^ for another study. Subjects were allowed to rest as long as needed in between walking trials. The median number of days in between the two measurement days was 5 (range 1–21). Subjects were asked to perform their normal activities on the day before each measurement day.

### Procedure

Upon arrival at the laboratory, each subject was first informed about the measurement procedure and then familiarized with treadmill walking. Subjects were allowed to practice treadmill walking for any amount of time. In general, subjects were comfortable with treadmill walking within 5 min. Subjects were instrumented with clusters of 3 LED’s on the trunk, at the level of T6, and on both feet. An optoelectronic system (Optotrak Northern Digital Inc., Waterloo, Ontario) measured the LED positions at 50 samples s^−1^.

### Gait measures

The extracted gait variability measures were variability of medio-lateral trunk center of mass velocity (VAR_ml_), stride-time-variability (VAR_ST_) and step-width-variability (VAR_SW_) of the final 150 strides of each trial (approximately the final 2–3 min). VAR_ml_ was calculated as the mean of the standard deviations of medio-lateral trunk velocities at each increment of normalized time (0–100 %) of the measured strides. Trunk center of mass position was estimated based on the position of the LED-cluster attached to the trunk, trunk circumference and the position of several bony landmarks relative to the cluster [19]. The data were low-pass filtered (20 Hz, second-order lowpass Butterworth), for gait variability measures only, before 3-point differentiation to obtain trunk velocities. VAR_ST_ was calculated as the standard deviation of the final 150 stride times. Stride time was calculated as the time between consecutive foot contacts of the same foot, which were determined as the local minima of the vertical position of the feet cluster markers. Step width was calculated as the maximal perpendicular distance relative to the walking direction between the lateral malleoli for each step. VAR_SW_ was calculated as the standard deviation of the final 300 steps.

Gait stability was quantified using local divergence exponents (LDE) [20]. LDEs describe how small initial differences in kinematics progress over the course of a step. The method for calculating the LDE has been described previously in more detail [16, 20]. In the present study, we used a reconstructed state-space based on a single time-series of medio-lateral trunk velocity and a state-space reconstructed from trunk kinematics in six degrees of freedom, to obtain LDE_ml_ and LDE_trunk_, respectively. Parameters for state space reconstruction were based on data-driven estimates of the appropriate time-delay using the average mutual information procedure and the required number of embedded dimensions using the global false nearest neighbor analysis. LDE_ml_ was determined from a 5-dimensional state-space from embedded medio-lateral trunk velocity time-series, with a delay of 10 samples. LDE_trunk_ was based on a 12-dimensional state space reconstructed by combining the 3-dimensional linear and angular velocities of the trunk and their time delayed copies. The embedding delay for this 12-dimensional state-space was 25 samples. Rosenstein’s algorithm was used to calculate the LDE [21] from the state space reconstructions. In short, for each time point in state-space, a nearest neighbor was found and the Euclidean distance between these points in state-space was tracked, resulting in a number of time–distance curves equal to the number of time points in state space. The divergence curve was then calculated as the mean of the natural log of the time–distance curves. Finally, the LDE was determined as the slope of the linear fit through the first 50 samples (time needed for one step on average) of the divergence curve, corresponding to the initial period of rapid exponential divergence. Thus, the LDE indicates the rate of logarithmic divergence as a result of differences in initial conditions over the time needed for one step. A positive LDE indicates local instability.

### Statistical analysis

As pointed out in the introduction, power calculations in gait studies require information about between-subjects and within-subjects variance components of the gait measures of interest, the latter including variances between measurement days and between trials within a day. All gait measures were obtained, as described above, in two separate trials on each of two different days for each subject. The parent data set, thus, consisted of 64 values for each gait measure (16 subjects × 2 days × 2 trials). These 64 values provided the basis for the analyses of variance and power, performed for each separate gait measure. A nested random model was used to estimate variance components [22], by solving expected mean squares of the two-way (subject, day) ANOVA corresponding to this model. This assumes that no systematic sources of variance (fixed effects) are present in the data. To check the validity of this assumption, a repeated-measures ANOVA was performed to test for effects of day (first vs second) and trial (first vs second, within day) on each of the gait measures. Neither day, trial nor their interaction had any systematic effect (*p* > 0.05, absolute differences <5 %).

The estimates obtained from the parent data were the overall mean (*m*) and three variance components: variance between subjects ($s_{\text{BS}}^{2}$), variance between days within subjects ($s_{\text{BD}}^{2}$), and variance between trials within days within subjects ($s_{\text{WD}}^{2}$). These parameters can be used to estimate the number of subjects required to obtain sufficient power for different measurement strategies as outlined in the “Appendix”. For all analyses, the desired level of significance was set to 0.05 and power was set to 0.80. Additional assumptions needed regard the correlation (*ρ*) between measurements in the two compared conditions (e.g., before and after an intervention) at the level of individuals, i.e., the predictability of the result in one condition from that in the other for any particular subject. As far as we know, such values have not been reported for gait measures in the literature. Therefore, we explored a range of values of *ρ* (0.3–0.6–0.9) as possible scenarios.

Based on these settings, we estimated the required number of subjects, *n*_s_, to detect effects of 10 and 30 % of the mean of the reference condition for repeated-measures (paired) designs, under the scenario that only one trial was performed by each subject in each condition. The detectable effect sizes were arbitrarily chosen, but are in the order of magnitude reported in the literature for comparisons between fallers and non-fallers [8–10, 23–25].

To answer the second research question, we evaluated how a change in the number of measurement days or trials per day would influence the required number of subjects at a maintained statistical power. One or 2 measurement days and 1–3 trials per day were selected as realistic measurement strategies in clinical gait studies.

To estimate the prediction intervals of the calculated distribution parameters in the parent data set (*m*, $s_{\text{BS}}^{2}$, $s_{\text{BD}}^{2}$, $s_{\text{WD}}^{2}$), and of the required numbers of subjects, we used a bootstrap technique [26, 27]. In short, sixteen subjects were randomly drawn with replacement from the original 16 subjects, keeping the results from the four trials of each of the 16 selected subjects. Thus, one resampled bootstrap data set contained the same number of subjects and trials as the parent data set. For the resampled data set, the mean and variance components (*m*, $s_{\text{BS}}^{2}$, $s_{\text{BD}}^{2}$, $s_{\text{WD}}^{2}$) as well as n_s_ were estimated for all combinations of number of days and number of trials. This procedure was repeated for 5000 bootstrap data sets, and bias-corrected 95 % prediction intervals for each of the estimated parameters were obtained from the distribution of the 5000 determinations as a measure of estimation uncertainty [28]. All statistical analyses were done in R 2.13 [29].

## Results

All three variance components, key factors for estimating the required numbers of subjects in any particular data collection strategy, were substantial (see Table 1). For the gait variability measures VAR_ST_, VAR_SW_, and VAR_ml_, between-subject variance was larger than within-subject variance. For LDE measures, the sum of the two within-subject variance components was similar to the between-subjects variance, and between-days variance was two to three times larger than within-day variance. All variance components had wide 95 % prediction intervals.

**Table 1:** Distribution parameters of gait measures

	Mean	$s_{\text{BS}}^{2}$	$s_{\text{BD}}^{2}$	$s_{\text{WD}}^{2}$
VAR_ST_	39.5 ms (34.2–47.3)	156.8 (17.0–407.0)	45.9 (8.0–106.4)	32.9 (18.4–57.1)
VAR_SW_	2.8 cm (2.5–3.4)	0.67 (0.09–1.6)	0.09 (0.005–0.21)	0.21 (0.13–0.30)
VAR_ml_	2.8 cm s^−1^ (2.6–3.1)	3.3e^−3^ (8.8e^−4^–7.5e^−3^)	6.3e^−4^ (3.6e^−5^–1.4e^−3^)	8.8e^−4^ (5.2e^−4^–1.4e^−3^)
LDE_ml_	1.7 (1.6–2.0)	0.17 (0.06–0.30)	0.09 (0.03–0.16)	0.04 (0.01–0.07)
LDE_trunk_	1.1 (1.1–1.3)	0.04 (0.01–0.07)	0.03 (0.01–0.04)	0.01 (0.00–0.03)

Mean value and variance components between subjects ($s_{\text{BS}}^{2}$), within subjects between days ($s_{\text{BD}}^{2}$), and within subjects and days within days ($s_{\text{WD}}^{2}$) for stride time variability, step width variability, variability of medio-lateral trunk velocity, and medio-lateral and trunk local divergence exponents. In brackets: 95 % prediction intervals, as derived from the bootstrap simulations

*VAR*_*ST*_ stride time variability, *VAR*_*SW*_ step width variability, *VAR*_*ml*_ variability of medio-lateral trunk velocity, *LDE*_*ml*_ the local divergence exponent of medio-lateral trunk velocity, *LDE*_*trunk*_ the local divergence exponent of trunk kinematics

The numbers of subjects required to obtain sufficient statistical power in studies collecting data from one trial on 1 day in each of the two compared conditions ranged from 7 to 13 for highly correlated (*ρ* = 0.9) data with a large effect (30 %), up to 78–192 for data with a low correlation (*ρ* = 0.3) and with a small effect (10 %; Table 2).

**Table 2:** Required numbers of subjects to detect differences of 10 and 30 % of the reference group mean value for repeated-measures (paired) research designs with different values of correlations between measurements within subjects (*ρ*)

	*n*_s_^a^					
	*ρ* = 0.3		*ρ* = 0.6		*ρ* = 0.9	
	Δ10 %^b^	Δ30 %^b^	Δ10 %^b^	Δ30 %^b^	Δ10 %^b^	Δ30 %^b^
VAR_ST_	192 (78–306)	24 (12–38)	145 (70–213)	18 (10–26)	98 (57–138)	13 (10–18)
VAR_SW_	151 (81–237)	19 (12–29)	113 (72–159)	15 (11–21)	74 (55–96)	11 (10–14)
VAR_ml_	78 (38–127)	11 (7–17)	58 (31–89)	9 (7–13)	39 (24–57)	7 (7–10)
LDE_ml_	119 (80–167)	15 (11–21)	95 (67–131)	13 (11–18)	70 (46–106)	10 (8–15)
LDE_trunk_	81 (59–108)	11 (9–15)	67 (49–90)	10 (9–14)	53 (35–76)	8 (7–11)

Results with 95 % prediction intervals in brackets, as obtained by bootstrap simulation, are shown for stride time variability, step width variability, variability of medio-lateral trunk velocity, and medio-lateral and trunk local divergence exponents. All results refer to a data collection strategy of one trial on 1 day per subject and measurement condition

*VAR*_*ST*_ stride time variability, *VAR*_*SW*_ step width variability, *VAR*_*ml*_ variability of medio-lateral trunk velocity, *LDE*_*ml*_ the local divergence exponent of medio-lateral trunk velocity, *LDE*_*trunk*_ the local divergence exponent of trunk kinematics

^a^Required numbers of subjects, each of which is measured in both compared conditions (e.g., before and after an intervention)

^b^Difference between conditions, expressed in percentage of the group mean value in the control condition, cf. Eq. (2) in “Appendix”

**Fig. 1:**
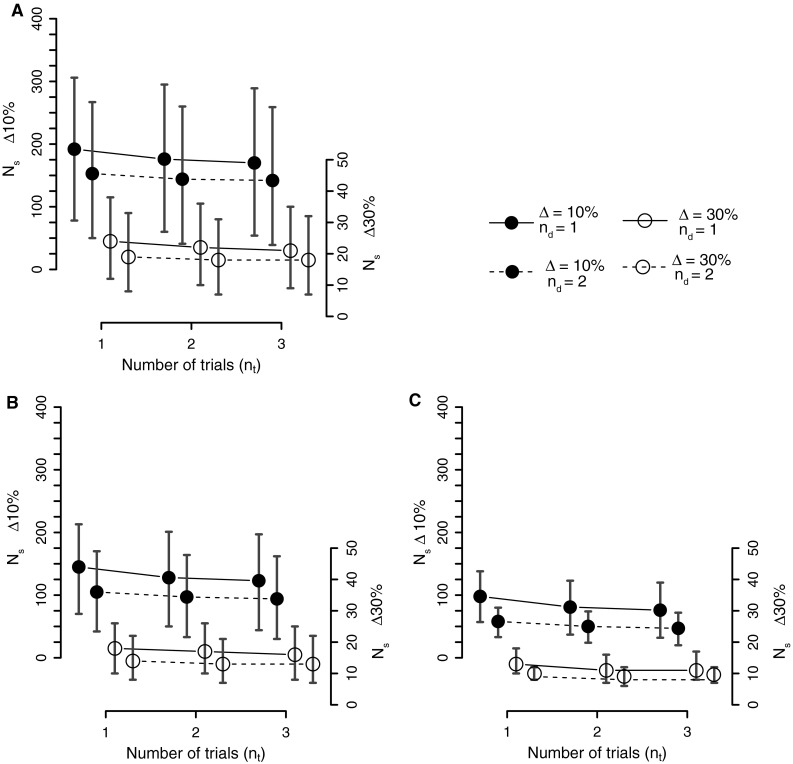
The required number of subjects to detect differences in stride time variability, VAR_ST_, between two conditions using different repeated-measures designs. The required numbers of subjects (each measured in both conditions) to detect a 10 % (*filled circles*, *left axis*) or 30 % (*unfilled circles*, *right axis*) change of VAR_ST_ in in paired designs with *ρ* = 0.3, 0.6, 0.9 (**b**, **c**, **d**, respectively). *Solid* and *dashed lines* indicate measurement strategies of 1 and 2 measurement days (*n*_d_ = 1 and *n*_d_ = 2), respectively. Results for one measurement day and one trial per day are identical to those shown in Table 2. *Error bars* show 95 % prediction intervals according to the bootstrap procedure

The effect of changing the measurement strategy on the required number of subjects is illustrated for VAR_ST_ in Fig. 1. Similar effects of changing the measurement strategy were obtained for the other gait measures. The largest decrease in the required numbers of subjects occurred when an additional measurement day was added. Conducting more trials on the same day did result in fewer required subjects, but it was generally less effective than increasing the number of measurement days, in particular when increasing the number of trials from two to three.

## Discussion

The main objective of this paper was to assess the numbers of subjects required to obtain sufficient statistical power (80 %) for detecting specified differences in gait measures between two conditions using subjects as their own controls, i.e., a repeated-measures design. In this study, we set the differences to 10 and 30 % of the mean value in the reference condition based on results reported in literature. These differences are in line with suggested meaningful changes reported by Brach et al. [30], i.e., 0.01 s for stance time and swing time variability and 0.25 cm for step length variability. These changes correspond to approximately 10 and 30 %, respectively, of the baseline mean value of these gait measures. However, more research on clinically relevant change in gait variability is warranted. To the best of our knowledge, there is no literature on meaningful or relevant changes of LDE. While we have exemplified calculation procedures and effects on study sizes using the 10 and 30 % differences, any other expected effects can be addressed using the data and equations presented in the paper and “Appendix”.

Regarding effects of physical training on gait variability, one small study [31] reported a large effect (35 %) and one large study a small (4 %) and non-significant effect [31]. To our best knowledge, no reports are available on effects of physical training on gait LDE. A meta-analysis on training effects on standing balance reported a small effect size, i.e. 11 % [32]. The results of the present study demonstrate that when expected differences are small, as illustrated by a 10 % change of the group mean, the required numbers of subjects is large (Table 2). Since a 10 % change, or even less, in gait measures between conditions might be clinically relevant [30], it is advisable to measure a large number of subjects and to report both significant and non-significant results of several gait measures to allow future meta-analyses.

The dominant cause of the need for large study sizes is the large gross between-subjects variance of gait measures, which in turn depends on the between-subjects variance and the variance associated with estimating a mean value of a gait measure in each subject. The latter affects the uncertainty associated with gait studies in its own right and also decreases the effective correlation between pairs of measurements (cf. “Appendix”). Like the clinically relevant effect sizes, the correlations between pairs of measurements before and after intervention, which quantify the predictability of the intervention result for any subject, are largely unknown. Van Schooten et al. [33] found correlations between conditions ranging from 0.55 to 0.97 for gait variability measures and LDE (personal communication). Hak et al. [34] found that the predictability of gait variability and stability measures varied with the effect size, small effects showing correlations from 0.33 to 0.79 and large effects showing correlations between −0.28 and 0.56 (personal communication). A conservative estimate of the correlation may therefore be justified. We tested different sizes of the “true”, error-free correlation between measurements in the pre- and post-intervention conditions in our analyses. From Fig. 1, it is clear that the correlation had a large influence on the required numbers of subjects. The error-free correlation is effectively reduced by the substantial within-subjects error associated with determining gait measures (see “Appendix”).

In the present study, we used treadmill walking at a fixed gait speed. Treadmill walking was used to allow collecting data from a large number of strides, to improve precision of estimates of gait variability [14, 15] and stability [16, 17]. In clinical practice, gait data is often collected in overground walking, using optoelectronic methods or electronic walkways, which limit data collection to a few strides. This increases within-subject variance and thus decreases statistical power to detect differences between groups and conditions. Data on larger numbers of strides can be collected in overground walking when using inertial sensors [35, 36], but the number of consecutive strides is usually still limited by spatial constraints. Therefore, as an alternative to collecting a large number of consecutive strides, the number of trials can be increased [37, 38]. It should be kept in mind that treadmill walking in itself affects gait variability and stability [39] and this may limit generalizability of the present results to overground walking, although statistical precision of stability estimates appears similar between overground [36, 38] and treadmill walking [18]. The fixed gait speed used, may have affected the between- and within-subjects variance components. However, since we did not establish preferred gait speeds, and since there is no consensus on the nature of the relationship between gait speed on the one hand and gait variability [40–44] and LDE [40, 41, 45–47] on the other hand, it is impossible to estimate the effect of gait speed on the results. Thus, generalization to studies using preferred speed should be done with care.

For VAR_ST_ and LDE_ml_ and LDE_trunk_, the between-days variance was higher than the within-day variance, but the between-days variance was also substantial for the other gait measures. Since subjects were exposed to similar conditions on both measurement days, the large between-day variances imply that other factors might influence the gait measures on a particular day. It could be that healthy subjects have a broad array of variability and LDE within which, for example, balance and agility are sufficient, and thus not further controlled. This could imply that a more challenging gait assessment, i.e., using mechanical and/or cognitive challenges to bring gait more toward the boundary of stable gait, is required to assess gait quality. The requirement to maintain global stability in such conditions might reduce the redundancy of gait performance and consequently reduce within-subject variance. In addition, more challenging test conditions, whether mechanical or cognitive, may increase effect sizes, much like these conditions often increase between-group differences in stability and variability [e.g. 48, 49]. However, decreased between-group differences under more challenging conditions have also been described [e.g., 50] and consequently the effect of using more challenging test conditions on statistical power of measurement strategies requires further study.

Our analysis of the effects of changing the number of measurements days per subject and trials per day clearly demonstrated that the former is more effective in reducing the number of required subjects than the latter, but that both have an effect. The large increase in statistical power when measuring subjects on multiple days is an effect of the generally large between-days variance, while within-day variances were, in general, smaller. It should be noted, though, that it will always be more beneficial to allocate multiple measurements to different days than to collect them on the same day, since this will more effectively reduce the gross between-subject variance (“Appendix”, Eq. 4).

Within-subject variance components as well as between-subject variance may be dependent on the subject group studied. The present study involved healthy and relatively young (mean age 65 years) older adults. Results can, thus, not be generalized to patient populations and older and potentially more frail elderly.

Calculations of LDE allow for many different choices of the number of embedding dimensions and time-delays when constructing the state-space. While it is most common to use a fixed dimensionality (5D or 12D) of the state-space, different approaches to estimate these parameters have also been used [51]. Furthermore, the region of the divergence curve used to estimate the slope also needs to be selected. We did not investigate the effects of these choices on statistical power of LDE in gait studies. However, a study on the effects of these choices on the reliability of LDE exponents demonstrated that a fixed state-space reconstruction is generally more reliable than an individualized approach [36].

The prediction intervals of variance components (Table 1) and thus of the required number of subjects (Table 2) were wide, in the latter case particularly when investigating small differences between conditions. Wide prediction intervals of variance components are in line with reports from a few studies assessing postures and muscle activity in occupational settings [27, 52]. These wide prediction intervals complicate the determination of the required numbers of subjects. It has been suggested to base the study size on the 80th percentile of the distribution of the required number of subjects (cf. Table 2) rather than on the point estimate, which is in general downward (“optimistically”) biased [53]. The wide prediction intervals also imply that a pilot study with a small number of subjects is not likely to result in reliable data for power calculations. An unreliable power analysis could lead to underpowered studies and hence a waste of time, effort, and money in executing a study that will probably be inconclusive, but it could also result in overpowered studies, which would, indeed, have a high probability of resulting in statistically significant findings, but also consume unnecessarily large resources in reaching these results.

## Conclusions

The results of the present study indicate that studies attempting to detect small changes in gait variability and stability between conditions measured in the same subjects (i.e., a repeated-measures design) need a large sample of subjects, generally well over 50, to obtain sufficient statistical power. To increase statistical power, increasing the number of measurement days is more effective than increasing the number of trials within a day. The presented results are important when interpreting studies that report small and non-significant effects.

## Appendix

The variance in the parent data set was partitioned using a nested random model [22]:

1 $$ {\text{GM}}_{\text{sdt}} = \mu + \alpha_{\text{s}} + \beta_{\text{sd}} + \varepsilon_{\text{sdt}} $$

where, GM_sdt_ is the value of the gait measure in trial *t*, collected on day *d* in subject *s*; *μ* is the group mean; *α*_s_ the effect of subject, *s* = 1, 2, … , 16; *β*_sd_ the effect of day within subject, *d* = 1,2; *ε*_sdt_ the residual corresponding to trial within day and subject, *t* = 1, 2.

Variance components were estimated by solving expected mean squares of the two-way nested ANOVA corresponding to Eq. (1). Thus, the parent data were used to estimate the overall mean (*m*, the estimate of *μ*) and the three variance components: variance between subjects ($s_{\text{BS}}^{2}$, the estimated variance of *α*_s_), variance between days within subjects ($s_{\text{BD}}^{2}$, the estimated variance of *β*_sd_), and variance between trials within days within subjects ($s_{\text{WD}}^{2}$, the estimated variance of *ε*_sdt_).

The required number of subjects to obtain sufficient statistical power to detect a significant difference between two conditions within subjects by means of a paired *t* test is given by:

2 $$ {n_{\text{s}} = \frac{{s_{\Delta }^{2} \times \left( {t_{{n_{s} - 1,1 - \beta }} + t_{{n_{\text{s}} - 1,1 - \alpha /2}} } \right)^{2} }}{{\Delta^{2} }}} $$

where *n*_*s*_ is the required number of subjects (each measured in both conditions); Δ the specified effect to be detected; $s_{{_{\Delta } }}^{2}$ the variance of the difference between conditions; *t*_*df*,*p*_ the *p* percentile of the *t* distribution with *df* degrees of freedom, 1 − *β* desired level of statistical power, and *α* desired level of significance. $s_{{_{\Delta } }}^{2}$ depends on the gross between-subjects variance ($s_{{_{\text{S}} }}^{2}$) and the adjusted correlation between conditions in the paired design (*ρ*′) as shown in Eq. (3):

3 $$ {s_{\Delta }^{2} = 2 \times s_{\text{S}}^{2} \times \left( {1 - \rho^{\prime } } \right)} $$

where $s_{{_{\text{S}} }}^{2}$ is the gross between-subjects variance, which in turn depends on the between-subjects variance ($s_{\text{BS}}^{2}$) and the variance associated with estimating a mean value of a gait measure in one subject according to:

4 $$ {s_{S}^{2} = s_{\text{BS}}^{2} + \frac{{s_{\text{BD}}^{ 2} }}{{n_{\text{d}} }} + \frac{{s_{\text{WD}}^{2} }}{{n_{\text{d}} \times n_{\text{t}} }}} $$

where *n*_d_ is number of measured days per subject and *n*_*t*_ are number of trials per day and subject.

In Eq. (3), *ρ*′ is the adjusted correlation between results obtained by a subject in the two compared conditions, i.e., an estimate of the predictability of the result in one condition from that in the other (e.g., the predictability of an intervention effect). *ρ*′ depends on the ratio of $s_{\text{BS}}^{2}$–$s_{{_{\text{S}} }}^{2}$:

5 $$ {\rho^{\prime } = \rho \times \frac{{s_{\text{BS}}^{2} }}{{s_{\text{S}}^{2} }}} $$

where *ρ* is the “true” within-subject correlation between measurements in the two compared conditions in the ideal case of error-free measurements.

Equation (2) has to be solved by iterative methods because *n*_s_ occurs on both sides of the equal sign.

## References

[1] Berg WP, Alessio HM, Mills EM (1997). Circumstances and consequences of falls in independent community-dwelling older adults. Age Ageing.

[2] Niino N, Tsuzuku S, Ando F (2000). Frequencies and circumstances of falls in the National Institute for Longevity Sciences, Longitudinal Study of Aging (NILS-LSA). J Epidemiol.

[3] Robinovitch SN, Feldman F, Yang Y (2013). Video capture of the circumstances of falls in elderly people residing in long-term care: an observational study. Lancet.

[4] Granacher U, Muehlbauer T, Gollhofer A (2011). An intergenerational approach in the promotion of balance and strength for fall prevention—a mini-review. Gerontology.

[5] Seidler RD, Bernard JA, Burutolu TB (2010). Motor control and aging: links to age-related brain structural, functional, and biochemical effects. Neurosci Biobehav Rev.

[6] Hamacher D, Singh NB, Van Dieen JH (2011). Kinematic measures for assessing gait stability in elderly individuals: a systematic review. J R Soc Interface R Soc.

[7] Bruijn SM, Meijer OG, Beek PJ (2013). Assessing the stability of human locomotion: a review of current measures. J R Soc Interface R Soc.

[8] Hausdorff JM, Rios DA, Edelberg HK (2001). Gait variability and fall risk in community-living older adults: a 1-year prospective study. Arch Phys Med Rehabil.

[9] Maki BE (1997). Gait changes in older adults: predictors of falls or indicators of fear. J Am Geriatr Soc.

[10] Toebes MJP, Hoozemans MH, Furrer R (2012). Local dynamic stability and variability of gait are associated with fall history in elderly subjects. Gait Posture.

[11] Weiss A, Brozgol M, Dorfman M (2013). Does the evaluation of gait quality during daily life provide insight into fall risk? A novel approach using 3-day accelerometer recordings. Neurorehabil Neural Repair.

[12] Rispens SM, van Schooten KS, Pijnappels M (2015). Identification of fall risk predictors in daily life measurements: gait characteristics’ reliability and association with self-reported fall history. Neurorehabil Neural Repair.

[13] van Schooten KS, Pijnappels M, Rispens SM (2015). Ambulatory fall risk assessment: quality and quantity of daily life gait predict falls in older adults. J Gerontol.

[14] Hollman JH, Childs KB, McNeil ML (2010). Number of strides required for reliable measurements of pace, rhythm and variability parameters of gait during normal and dual task walking in older individuals. Gait Posture.

[15] Owings TM, Grabiner MD (2003). Measuring step kinematic variability on an instrumented treadmill: how many steps are enough?. J Biomech.

[16] Bruijn SM, van Dieen JH, Meijer OG (2009). Statistical precision and sensitivity of measures of dynamic gait stability. J Neurosci Methods.

[17] Kang HG, Dingwell JB (2006). Intra-session reliability of local dynamic stability of walking. Gait Posture.

[18] Reynard F, Terrier P (2014). Local dynamic stability of treadmill walking: intrasession and week-to-week repeatability. J Biomech.

[19] Zatsiorsky V (2002). Kinetics of human motion.

[20] Dingwell JB, Cusumano JP (2000). Nonlinear time series analysis of normal and pathological human walking. Chaos.

[21] Rosenstein MT, Colling JJ, DeLuca CJ (1993). A practical method for calculating largest Lyapunov exponents from small data sets. Phys D.

[22] Searle SR, Casella G, McCulloch CE (1992). Variance Components.

[23] Barak Y, Wagenaar RC, Holt KG (2006). Gait characteristics of elderly people with a history of falls: a dynamic approach. Phys Ther.

[24] Hausdorff JM, Edelberg HK, Mitchell SL (1997). Increased gait unsteadiness in community-dwelling elderly fallers. Arch Phys Med Rehabil.

[25] Paterson K, Hill K, Lythgo N (2011). Stride dynamics, gait variability and prospective falls risk in active community dwelling older women. Gait Posture.

[26] Diaconis P, Efron B (1983). Computer-intensive methods in statistics. Sci Am.

[27] Mathiassen SE, Burdorf A, van der Beek AJ (2002). Statistical power and measurement allocation in ergonomic intervention studies assessing upper trapezius EMG amplitude. A case study of assembly work. J Electromyogr Kinesiol.

[28] Efron B, Tibshirani R (1986). Bootstrap methods for standard errors, confidence intervals, and other measures of statistical accuracy. Stat Sci.

[29] Development Core Team R (2011). R: A language and environment for statistical computing.

[30] Brach JS, Perera S, Studenski S (2010). Meaningful change in measures of gait variability in older adults. Gait Posture.

[31] Granacher U, Muehlbauer T, Bridenbaugh S (2010). Balance training and multi-task performance in seniors. Int J Sports Med.

[32] Latham NK, Bennett DA, Stretton CM (2004). Systematic review of progressive resistance strength training in older adults. J Gerontol.

[33] Van Schooten KS, Sloot LH, Bruijn SM (2011). Sensitivity of trunk variability and stability measures to balance impairments induced by galvanic vestibular stimulation during gait. Gait Posture.

[34] Hak L, Houdijk H, Steenbrink F (2012). Speeding up or slowing down?: gait adaptations to preserve gait stability in response to balance perturbations. Gait Posture.

[35] Bruijn SM, Ten Kate WR, Faber GS (2010). Estimating dynamic gait stability using data from non-aligned inertial sensors. Ann Biomed Eng.

[36] van Schooten KS, Rispens SM, Pijnappels M (2013). Assessing gait stability: the influence of state space reconstruction on inter- and intra-day reliability of local dynamic stability during over-ground walking. J Biomech.

[37] Kressig RW, Beauchet O (2006). Guidelines for clinical applications of spatio-temporal gait analysis in older adults. Aging Clin Exp Res.

[38] van Schooten KS, Rispens SM, Elders J (2013). Towards ambulatory balance assessment: estimating variability and stability from short bouts of gait. Gait Posture.

[39] Dingwell JB, Cusumano JP, Cavanagh PR (2001). Local dynamic stability versus kinematic variability of continuous overground and treadmill walking. J Biomech Eng.

[40] Dingwell JB, Marin LC (2006). Kinematic variability and local dynamic stability of upper body motions when walking at different speeds. J Biomech.

[41] Bruijn SM, van Dieen JH, Meijer OG (2009). Is slow walking more stable?. J Biomech.

[42] Jordan K, Challis JH, Newell KM (2007). Walking speed influences on gait cycle variability. Gait Posture.

[43] Moe-Nilssen R, Helbostad JL (2005). Interstride trunk acceleration variability but not step width variability can differentiate between fit and frail older adults. Gait Posture.

[44] Yamasaki M, Sasaki T, Torii M (1991). Sex difference in the pattern of lower limb movement during treadmill walking. Eur J Appl Physiol.

[45] England SA, Granata KP (2007). The influence of gait speed on local dynamic stability of walking. Gait Posture.

[46] Kang HG, Dingwell JB (2008). Effects of walking speed, strength and range of motion on gait stability in healthy older adults. J Biomech.

[47] Buzzi UH, Ulrich BD (2004). Dynamic stability of gait cycles as a function of speed and system constraints. Mot Control.

[48] Beurskens R, Wilken JM, Dingwell JB (2014). Dynamic stability of individuals with transtibial amputation walking in destabilizing environments. J Biomech.

[49] Lamoth CJ, van Deudekom FJ, van Campen JP (2011). Gait stability and variability measures show effects of impaired cognition and dual tasking in frail people. J Neuroeng Rehabil.

[50] Lamoth CJ, Ainsworth E, Polomski W (2010). Variability and stability analysis of walking of transfemoral amputees. Med Eng Phys.

[51] Gates DH, Dingwell JB (2009). Comparison of different state space definitions for local dynamic stability analyses. J Biomech.

[52] Liv P, Mathiassen SE, Svendsen SW (2012). Accuracy and precision of variance components in occupational posture recordings: a simulation study of different data collection strategies. BMC Med Res Methodol.

[53] Browne RH (1995). On the use of a pilot sample for sample size determination. Stat Med.

